# Inhibitory Effect of Protease Inhibitors on Larval Midgut Protease Activities and the Performance of *Plutella xylostella* (Lepidoptera: Plutellidae)

**DOI:** 10.3389/fphys.2018.01963

**Published:** 2019-01-15

**Authors:** Aiping Zhao, Yin Li, Chunmeng Leng, Ping Wang, Yiping Li

**Affiliations:** ^1^Key Laboratory of Plant Protection Resources and Pest Management, Ministry of Education, Northwest A&F University, Yangling, China; ^2^State Key Laboratory of Crop Stress Biology for Arid Areas, Northwest A&F University, Yangling, China; ^3^Department of Entomology, Cornell University, Ithaca, NY, United States

**Keywords:** *Plutella xylostella*, protease activity, protease inhibitors and activators, growth and development, midgut

## Abstract

*Plutella xylostella* L. (diamondback moth) is a pest of cruciferous plants. To understand the relationship among protease inhibitors, protease activities and the growth and development of this insect, the activities of midgut proteases of *P. xylostella* larvae were determined in this study. Protease samples were extracted from the midguts of *P. xylostella* larvae, and the protease activities were determined using enzyme specific substrates. The results showed that CaCl_2_, EDTA, and EGTA inhibited only the trypsin. Among the common protease inhibitors, phenylmethyl sulfonyl fluorine (PMSF), Nα-p-methyl sulfonyl-L-lysine chloromethylketone (TLCK), Nα-methyl sulfonyl-L- phenylalanine chloromethyl ketone (TPCK), soybean trypsin inhibitor (STI), and PMSF inhibited the total protease, high-alkaline trypsin (a trypsin subtype with highly alkaline pH optimum), low-alkaline trypsin (another trypsin subtype with slightly alkaline pH optimum), and chymotrypsin; TLCK inhibited the total protease and high-alkaline trypsin, whereas TPCK only activated the high-alkaline trypsin activities. STI had an inhibitory effect on all the proteases. These results showed that protease inhibitors had a certain extent inhibition to protease activities in the larval midgut of *P. xylostella* and that STI can potentially be used for effective pest control. The development of *P. xylostella* was delayed in the presence of different inhibitors. These effects were also related to the concentration of the inhibitor. A higher STI concentration showed a longer lasting effect but lower effect in this study compared to that of TLCK. The protease inhibitors had some inhibitory effect on the synthesis and secretion of proteases, and interfered with the protease activity, thereby inhibiting the absorption of nutrients and delaying the growth and development of *P. xylostella* and reducing their ability to reproduce. These findings should provide the baseline information about using for effective pest management in the future.

## Introduction

The midgut of insects contains proteases that are involved in several physiological, biochemical processes and promote food digestion and nutrient absorption. Proteases can be divided into four groups, namely serine proteases, metalloproteinases (MMPs), cysteine proteases and aspartate proteases. The serine proteases are mainly involved in digestive processes. Serine proteases especially trypsin, chymotrypsin and elastase in particular have a digestive function, i. e., they can break peptide bonds in large proteins to generate smaller peptides (Jiang and Kanost, 2000; Li et al., 2004). Most serine proteases have an active serine residue at a conserved position (Ser-195). In lepidopteran species, the larval midgut was reported to contain serine proteases, particularly trypsin and chymotrypsin (Milne and Kaplan, 1993; Srinivasan et al., 2006). The midgut of lepidopteran larvae has an alkaline environment where serine proteases are reported to have a high level of activity (Berenbaum, 1980; Pritehett et al., 1981; Applebaum, 1985), however, the optimum pH for trypsin activity varies across insect species (Broadway, 1989; Kipgen and Aggarwal, 2014; Zhao et al., 2016).

Protease inhibitors can prevent the protease activity by binding to the active sites or allosteric sites of proteases or their zymogens (Xiao et al., 2004). For developing insect-resistant plants using these inhibitors with the help of advanced technologies, such as genetic engineering, it is essential to have a thorough understanding about such inhibitors. Protease inhibitors can interfere with the synthesis and secretions of midgut proteases, and thereby, inhibit their trophic physiological function, growth and development (Hegedus et al., 2003; Chougule et al., 2005; Wu et al., 2013). The effects of some trypsin inhibitors on the growth of *Heliothis zea* (Boddie) have been examined (Broadway and Duffey, 1986). Few studies on the use of serine protease inhibitors for controlling herbivorous insects indicate that protease inhibitors can be employed in devising feasible and effective pest control strategies (Sagili et al., 2005; Tamhane et al., 2007).

*Plutella xylostella* L. (Lepidoptera: Plutellidae; diamondback moth) is a pest of cruciferous plants. There are several reports on the growth and development of *P. xylostella*, however, to date, only a few studies have examined the correlation between protease inhibitors and protease activities in the midgut of *P. xylostella*, and its effect on the growth and development of this insect. In the present study, we determined the midgut protease activity in the midgut of *P. xylostella* larvae fed a diet containing different protease inhibitors and examined the effect of protease inhibitors on *its* growth and development. The findings from this study could provide the foundation for establishing a new approach of effectively using protease inhibitors to control populations of this insect pest and other lepidopteran pests.

## Materials and Methods

### Insects

For this study, *P. xylostella* samples were obtained from a laboratory colony maintained at the College of Plant Protection, Northwest A&F University, Yangling, Shaanxi, China. The larvae were reared on *Brassica oleracea* (cv. Qingan 70) at 24 ± 2°C, 70 ± 10% relative humidity (RH) and under 15-h light:9-h dark photoperiod. The adults were provided with 10% honey solution for supplementary adult feeding to improve the female oviposition.

### Extraction of *P. xylostella* Midgut Proteases

The larval midgut proteases were extracted as described by Wang and Qin (1996). Healthy late 3^rd^ instar larvae of equal sizes were rapidly dissected on ice to collect the midgut, and the gut contents were flushed using 0.15 mol/L NaCl. Subsequently, the midgut was placed in a 1.5 mL centrifuge tube and rapidly homogenized over ice. The crude extract was centrifuged at 12,000 ×*g* for 15 min at 4°C, and the supernatant was collected and stored at -20°C. Protein concentration in the extract was assayed using the method of Bradford (1976).

### Determination of Protease Activity in the Midgut of *P. xylostella*

Total protease activity was determined as follows: A 20 mg/mL solution of azocasein, the substrate, was prepared in NaCl (0.15 mol/L) and stored at 4°C. Subsequently, the following substances were added to a 1.5 mL centrifuge tube: 100 μL azocasein solution, 10 μL midgut protease extract and 40 μL glycine/NaOH reaction buffer (0.1 mol/L, pH 11.0). The mixture was incubated at 30°C for 3 h, and then 150 μL of 20% (v/v) trichloroacetic acid (pre-cooled) was added to terminate the reaction. The mixture was centrifuged at 12,000 × *g* at 4°C for 15 min to collect the supernatant, which was termed as the midgut protease extract in this study. Protease activity in the extract was determined by measuring absorbance at 415 nm using a plate reader (Wang and Qin, 1996).

The trypsin activity was determined using two specific substrates: BAρNA (Nα-Benzoyl-DL-arginine-p-nitroanilide) and TAME (Nα-P-Tosyl-L-arginine methyl ester hydrochloride). BAρNA was dissolved in dimethyl sulfoxide (DMSO) at a concentration of 20 mg/mL and stored at 4°C. Then, 100 μL BAρNA, 10 μL midgut enzyme extract and 90 μL Tris-HCl reaction buffer (0.1 mol/L, pH 10.5) were added to a 1.5 mL centrifuge tube and incubated at 30°C for 20 min. Subsequently, 100 μL of 20% (v/v) trichloroacetic acid (pre-cooled) was added to terminate the reaction. The reaction mix was centrifuged and 200 μL of the supernatant was used to determine the absorbance at 405 nm using a plate reader. TAME was dissolved in NaCl (0.15 mol/L) at a concentration of 2 nmol/L. Then, 100 μL TAME, 10 μL midgut enzyme extract and 90 μL Tris-HCl reaction buffer (0.1 mol/L, pH 8.5) were added to a 1.5 mL centrifuge tube and incubated at 30°C for 20 min. The mixture was centrifuged, and the supernatant was used to measure the absorbance at 247 nm using a plate reader (Wirnt, 1974).

The chymotrypsin activity was determined using the substrate BTEE (substrate, N-Benzoyl-L-tyrosine ethyl ester) dissolved in NaCl (0.15 mol/L) at a concentration of 1 mmol/L and stored at 4°C. Then, in a 1.5 mL centrifuge tube, 100 μL BTEE solution, 10 μL midgut enzyme extract and 90 μL glycine/NaOH reaction buffer (0.1 mol/L, pH 9.0) were added and incubated at 30°C for 20 min and then centrifuged. An aliquot of the supernatant was used to measure absorbance at 256 nm using a plate reader (Wirnt, 1974).

### Effects of Protease Activators and Inhibitors on *P. xylostella* Larval Midgut Protease Activity

Four protease activators (MgCl_2_, CaCl_2_, EDTA and EGTA) and a total of six protease inhibitors IAA (In Alien Attitude), DTT (DL-Dithiothreitol), PMSF, TPCK, TLCK and STI (Soybean Trypsin Inhibitor) were used for these analyses. First, *P. xylostella* larval midgut protease extracts were obtained, and the effects of protease activators and inhibitors on the intestinal protease activity of *P. xylostella* larvae were determined. Each treatment had three replicates. The activity of the total protease was determined in a glycine/NaOH reaction buffer (0.1 mol/L, pH 11.0), that of high-alkaline trypsin enzyme (a trypsin subtype in the *P. xylostella* larval midgut protease extract having highly alkaline pH optimum) was determined in a Tris-HCl reaction buffer (0.1 mol/L, pH 10.5), low-alkaline trypsin (another trypsin subtype in the *P. xylostella* larval midgut protease extract having slightly alkaline pH optimum) was determined in a Tris-HCl reaction buffer (0.1 mol/L, pH 8.5), and that of chymotrypsin activity was measured in a glycine/NaOH reaction buffer (0.1 mol/L, pH 9.0; Zhao et al., 2017).

Each of the protease activators or inhibitors (10 μL) was mixed with 10 μL of the insect midgut enzyme extract and incubated at 30°C for l5 min. The corresponding substrate was added to measure the protease activity. Protease activity was determined. Double-distilled water (ddH_2_O) served as the control and the reaction was performed in triplicates for each treatment.

### Effect of Protease Inhibitors on the Protease Activities of *P. xylostella* Larvae

The leaf immersion method (Guo et al., 2013) was used for introducing protease inhibitors: cabbage (cv. Qingan 70) leaves were immersed in solutions containing the protease inhibitors, TPCK (2 mmol/L), TLCK (2 mmol/L) and STI (100 μg/mL) for 10 s and were then dried. The 3rd instar *P. xylostella* larvae were starved for 4 h and then were allowed to feed on the inhibitor-soaked cabbage leaves. Double distilled water served as the control. Midgut samples were collected at 0 (starved for 4 h), 4, 8, 12, 24, 36, 48, 60, 72, and 84 h to extract the enzymes, which were then stored at -20°C. For each treatment comprised 30 larvae were used in each replicate and there were three replicates per treatment. The protease activity was determined as described above.

### Effect of Different Protease Inhibitors on the Performance of *P. xylostella*

Cabbage (cv. Qingan 70) leaf sections (circular, 9 cm in diameter) were immersed for 10 s in solutions containing one of the three protease inhibitors: TPCK (2 mmol/L), TLCK (2 mmol/L) and STI (100 μg/mL, 50 μg/mL, 10 μg/mL)and were then dried. Double distilled water was used as the control. In each treatment, one hundred newly oviposited eggs were placed in Petri dishes (circular, plastic, 9 cm in diameter) containing the inhibitor-treated leaves. The lids were closed and sealed. The leaves were changed every second day. The time was recorded when an egg hatched. Each neonate larva was transferred to another Petri dish (circular, plastic, 9 cm in diameter) and numbered. When a larva transformed into a pupa, the time was recorded to calculate the larval duration was recorded. For each treatment, 30 larvae were used in each replicate and there were three replicates per treatment. The duration of the growth and developmental stages (larval, pupal, adult and larval-adult), the time of larval survival, pupal weight, and time of emergence were recorded and the average of each treatment, for example, pupation rate, larval survival rate, emergence rate for each treatment were calculated. A newly emerged male and female moth were put together in paper cups and fed with 10% honey water. The number of eggs and the time of death were recorded at 0800 and 2000 h daily until the emergence ceased.

### Data Analyses

The statistical significance of the differences between the control and treatment groups were analyzed by ANOVA (analysis of variance) with alpha = 0.05, 0.01 and 0.001, and respectively. The means were separated using Tukey’s HSD test, which are denoted with one (^∗^), two (^∗∗^), or three asterisks (^∗∗∗^), corresponding to *P*-values are less than 0.05, 0.01 or 0.001. SPSS 20.0 was used for statistical analyses, and GraphPad Prism 5 was used to create the figures.

## Results

### Effects of Protease Activators and Inhibitors on the Midgut Protease Activity of *P. xylostella* Larvae

**FIGURE 1:**
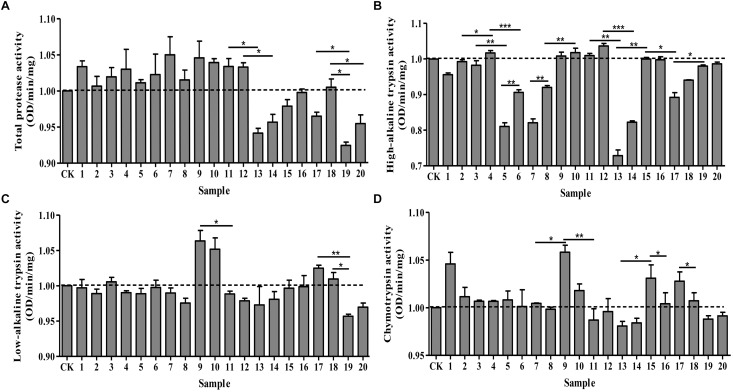
Effects of protease activators and inhibitors on the activity of proteases in *Plutella xylostella* larvae. **(A)** Total protease. **(B)** High-alkaline trypsin. **(C)** Low-alkaline trypsin. **(D)** Chymotrypsin. The dotted line indicates the control group. Columns represent means of three independent values and their SEM. CK (control): ddH_2_O; 1: 10 mmol/L CaCl_2_; 2: 5 mmol/L CaCl_2_; 3: 10 mmol/L MgCl_2_; 4: 5 mmol/L MgCl_2_; 5: 10 mmol/L EGTA; 6: 5 mmol/L EGTA; 7: 10 mmol/L EDTA; 8: 5 mmol/L EDTA; 9: 10 mmol/L IAA; 10: 5 mmol/L IAA; 11: 10 mmol/L DTT; 12: 5 mmol/L DTT; 13: 10 mmol/L PMSF; 14: 5 mmol/L PMSF; 15: 2 mmol/L TPCK; 16: 1 mmol/L TPCK; 17: 2 mmol/L TLCK; 18: 1 mmol/L TLCK; 19: 100 μg/mL STI; 20: 10 μg/mL STI. ^∗^*P* < 0.05, ^∗∗^*P* < 0.01, and ^∗∗∗^*P* < 0.001.

Under optimal pH, we determined the effects of protease activators and inhibitors on the midgut protease activity of *P. xylostella*. Different effects were observed on the activities of total protease, high-alkaline trypsin, low-alkaline trypsin and chymotrypsin (Figure 1). Total protease activity remained similar for the ddH_2_O control and the groups treated with MgCl_2,_ CaCl_2_, EGTA, EDTA, IAA and DTT. However, there were significant differences between the control and the remaining treatment groups: PMSF, TPCK, TLCK (1 mmol/L) and STI inhibited the activity of total proteases (*F* = 6.72, df = 20, 42, *P* < 0.05) (Figure 1A). Moreover, these inhibitory effects were concentration-dependent; higher concentrations resulted in greater inhibitory effects. On the other hand, the activity of high-alkaline trypsin (*F* = 98.52, df = 20, 42, *P* < 0.05) was activated by IAA, DTT, TPCK and 5 mmol/L MgCl_2_, while TPCK had no effect on it, the remaining treatments had an inhibitory effect on the activity (Figure 1B). IAA and TLCK activated the activity of low-alkaline trypsin (*F* = 5.78, df = 20, 42, *P* < 0.05), whereas TPCK had no effect on low-alkaline trypsin, and the other treatments had an inhibitory effect on the activity (Figure 1C). Lastly, the chymotrypsin (*F* = 4.96, df = 20, 42, *P* < 0.05) activity was inhibited by DTT, PMSF and STI, and the other treatments had an activating effect on the activity (Figure 1D). Overall, all the effects were concentration-dependent with higher concentrations of inhibitors resulting in maximum effects.

### Effects of Different Dietary Protease Inhibitors on the Midgut Protease Activity of *P. xylostella* Larvae

**FIGURE 2:**
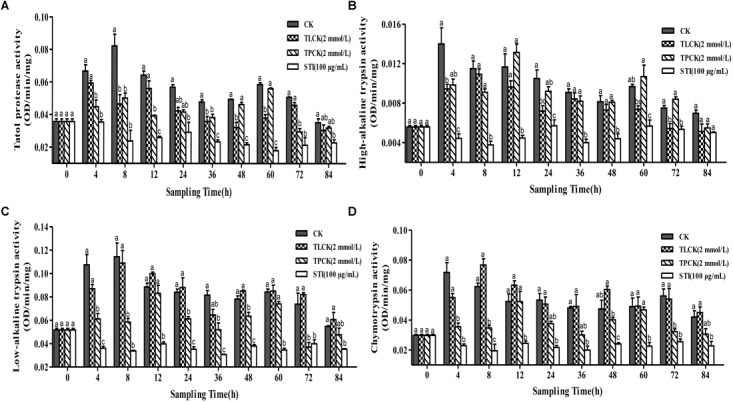
Effects of ingesting different protease inhibitors, TLCK, TPCK, and STI, on the midgut protease activity of *Plutella xylostella* larvae. **(A)** Total protease. **(B)** High-alkaline trypsin. **(C)** Low-alkaline trypsin. **(D)** Chymotrypsin. Columns represent means of three independent values and their SEM. Different lowercase letters indicate significant difference at *P* < 0.05 using Tukey’s HSD test.

The ability of protease inhibitors to inhibit larval tryptic and chymotryptic activity was further examined using the protease inhibitors, TPCK (2 mmol/L), TLCK (2 mmol/L) and STI (100 μg/mL) at different time points after feeding these to *P. xylostella* larvae. Overall, the inhibitory effects of the different protease inhibitors significantly differed over time (Figure 2). The inhibitory effect of STI on all proteases was the lowest among all the inhibitors. In general, all the inhibitors reduced the activity of the total protease in comparison to the control group. The total protease (*F* = 14.02, df = 3, 36, *P* < 0.05) activity of *P. xylostella* larvae that ingested the inhibitors in the control and the TPCK-fed groups first increased and then decreased during the 0–8 h period. The STI treatment had fluctuating effects on total protease activity between 0 and 4 h after of the treatment (Figure 2A). TLCK and STI inhibited the activity of high-alkaline trypsin (*F* = 10.48, df = 3, 36, *P* < 0.05), while TPCK occasionally inhibited the activity of high-alkaline trypsin (Figure 2B). TPCK and STI also inhibited the activity of low-alkaline trypsin (*F* = 19.79, df = 3, 36, *P* < 0.05) (Figure 2C). Moreover, TPCK and STI inhibited the activity of chymotrypsin (*F* = 22.70, df = 3, 36, *P* < 0.05), whereas TLCK did not inhibit the activity of chymotrypsin except between 0 and 4 h (Figure 2D). In the control group, the activity of the total protease was not constant, there are fluctuations, the same goes for other groups, and the frequency of fluctuations were different, in other words, the inhibitors disrupted the enzyme system of *P. xylostella*.

### Effects of Different Dietary Concentrations of STI on *P. xylostella* Larval Midgut Protease Activity

**FIGURE 3:**
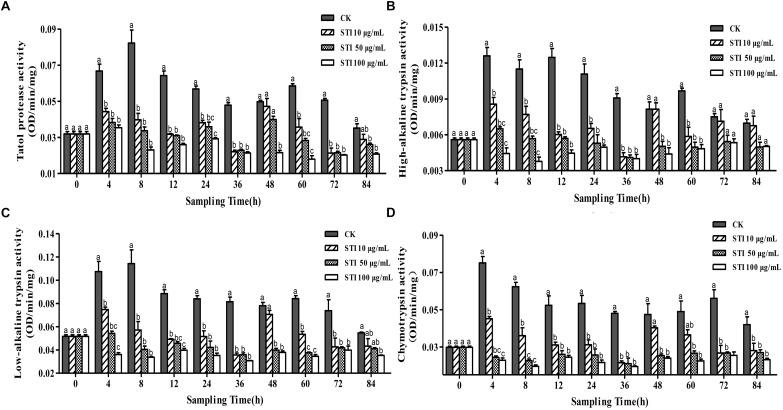
Effects of ingesting different concentrations of the protease inhibitor, STI, on the midgut protease activity of *Plutella xylostella* larvae. **(A)** Total protease. **(B)** High-alkaline trypsin. **(C)** Low-alkaline trypsin. **(D)** Chymotrypsin. Columns represent means of three independent values and their SEM. Different lowercase letters indicate significant difference at *P* < 0.05 level using Tukey’s HSD test.

When *P. xylostella* larvae were fed with four different concentrations STI, i. e. 100, 50, 10, and 0 μg/mL, the activities of total protease, high-alkaline trypsin, low-alkaline trypsin and chymotrypsin were altered (Figure 3). Overall, after 4 h, STI increased the inhibitory activity of the total protease (*F* = 17.85, df = 3, 36, *P* < 0.05), with the highest STI concentration producing the greatest effect (Figure 3A). In the control group, two peaks appeared at 8 and 60 h, the larvae gone into the next instar, the activity intensified, and the fluctuation frequency of the total protease activity were less than that in other groups, whereas in the groups treated with the other inhibitors four peaks appeared at 4, 24, 48 and 84 h. The inhibitory effect was the highest at 36 h. The interactions between the midgut protease and the inhibitors changed the activity of the midgut protease frequently. At 4 h, the inhibitory effect on the high-alkaline trypsin (*F* = 21.63, df = 3, 36, *P* < 0.05) activity was the highest with 100 μg/mL STI and lowest with 10 μg/mL STI (Figure 3B). The low-alkaline trypsin (*F* = 25.85, df = 3, 36, *P* < 0.05) activity in the control and 10 μg/mL STI groups increased, while there was no effect in the 50 μg/mL STI group, and the activity declined in the 100 μg/mL STI group (Figure 3C). In the treatment groups, there were three activity peaks at 4, 24 and 48 h with the lowest chymotrypsin (*F* = 31.80, df = 3, 36, *P* < 0.05) activity at 36 h. At 4 h the activities in the 100 μg/mL and 50 μg/mL STI groups were lower than those in the 100 μg/mL STI and the control groups (Figure 3D). At 36 h, the high-alkaline trypsin activity was the lowest among the three treatment groups.

### Effect of Dietary Protease Inhibitors on the Performance of *P. xylostella*

*Plutella xylostella* larvae were fed the protease inhibitors TPCK, TLCK and STI, and their growth and development were observed (Table 1). The larval duration (*F* = 3.62, df = 5, 12, *P* < 0.05), pupal duration (*F* = 2.77, df = 5, 12, *P* < 0.05), adult duration (*F* = 2.32, df = 5, 12, *P* < 0.05), larva-adult duration (*F* = 6.71, df = 5, 12, *P* < 0.05) of *P. xylostella* fed different protease inhibitors were significantly different. The larval-adult treatment duration were all longer than that of the control treatments and were affected by the concentration of the inhibitor, with higher STI concentrations having the longer duration.

**Table 1:** Developmental duration of *Plutella xylostella* ingested different protease inhibitors.

Treatments	Larval duration(d)	Pupal duration(d)	Adult duration(d)	Larva-adult duration(d)
CK	8.99 ± 0.32 c	4.54 ± 0.19 b	13.65 ± 0.28 b	27.18 ± 0.61 b
TPCK(2 mmol/L)	9.46 ± 0.28 ab	4.79 ± 0.12 ab	14.25 ± 0.29 a	28.50 ± 0.55 a
TLCK(2 mmol/L)	9.47 ± 0.30 ab	4.84 ± 0.06 a	13.84 ± 0.15 ab	28.16 ± 0.45 a
STI(100 μg /mL)	9.76 ± 0.15 a	4.93 ± 0.27 ab	14.31 ± 0.34 a	29.00 ± 0.28 a
STI(50 μg /mL)	9.50 ± 0.12 ab	4.79 ± 0.13 ab	13.95 ± 0.45 ab	28.24 ± 0.61 a
STI(10 μg /mL)	9.16 ± 0.30 bc	4.59 ± 0.07 b	13.76 ± 0.08 ab	27.30 ± 0.09 b


*Data in the table are means ± SE. Different lowercase letters in the same column indicate significant difference at P < 0.05 level as assessed by Tukey’s HSD test.*

The larval survival rate (*F* = 2.53, df = 5, 12, *P* < 0.05), pupation rate (*F* = 4.39, df = 5, 12, *P* < 0.05), pupal weight (*F* = 1.81, df = 5, 12, *P* < 0.05) and number of eggs per female (*F* = 5.51, df = 5, 12, *P* < 0.05) of *P. xylostella* were significantly different among treatments (Table 2). Overall, the pupation rate, pupal weight, and the number of eggs per female for all the treatment groups were lower than those for the control group. The concentration of the inhibitor influenced the emergence rate with higher concentrations resulting in a higher inhibitory effect. The larval survival rate and number of eggs per female are two important parameters affecting the population dynamics of *P. xylostella* fed 100 μg/mL. The mean larval survival rate of *P. xylostella* ingested TLCK and STI (100 μg/mL) were 89.17 and 86.71%, respectively, and the number of eggs per female were 112.07 and 120.17, respectively, which were lower than the mean larval survival and the number of eggs per female for the control group.

**Table 2:** Effects of ingesting different protease inhibitors on growth and development of *Plutella xylostella*.

Treatments	Larval survival rate(%)	Pupation rate(%)	Pupal weight(mg)	Emergence rate(%)	Number of eggs per female
CK	91.99 ± 0.77 ab	98.33 ± 2.89 a	5.75 ± 0.24 a	92.10 ± 0.17 a	142.67 ± 6.41 a
TPCK(2 mmol/L)	95.43 ± 3.19 a	88.33 ± 5.16 b	5.04 ± 0.37 b	89.10 ± 2.80 a	114.83 ± 5.25 b
TLCK(2 mmol/L)	89.17 ± 3.63 b	93.72 ± 1.99 ab	5.37 ± 0.52 ab	81.62 ± 3.62 a	112.07 ± 11.29 b
STI(100 μg/mL)	86.71 ± 2.92 b	87.69 ± 2.23 b	5.16 ± 0.08 ab	83.27 ± 6.77 a	120.17 ± 12.69 b
STI(50 μg/mL)	90.95 ± 3.72 ab	92.10 ± 2.71 b	5.26 ± 0.19 ab	87.20 ± 3.81 a	127.87 ± 9.97 ab
STI(10 μg/mL)	91.79 ± 3.86 ab	93.16 ± 3.35 ab	5.63 ± 0.49 ab	88.34 ± 5.48 a	137.33 ± 6.43 a


*Data in the table are means ± SE. Different lowercase letters in the same column indicate significant difference at P < 0.05 level as assessed by Tukey’s HSD test.*

## Discussion

The diverse protease inhibitors and activators had different effects on the activities of total protease, high-alkaline trypsin, low-alkaline trypsin and chymotrypsin in the midgut of *P. xylostella* larvae midgut. For example, EDTA inhibited all the four proteases, and EGTA activated only the high-alkaline trypsin. Some divalent metal ions (Ca^2+^, Mg^2+^) are activators of a variety of proteases (Wang and Qin, 1996). Although Ahmad et al. (1980) reported that Mg^2+^ and Ca^2+^ had no effect on the activity of these four enzymes, the current study demonstrates that CaCl_2_ inhibited the trypsin activity. Among the common protease inhibitors, such as PMSF, TLCK, TPCK and STI, PMSF had no effect on chymotrypsin but inhibited the total protease, high-alkaline trypsin, and low-alkaline trypsin; TLCK inhibited the total protease and high-alkaline trypsin, whereas, TPCK activated high-alkaline trypsin. The inhibitory effect of STI on all the proteases was the greatest among the four inhibitors examined. This suggests that ability of STI to form a bonds with the insect enzymes is strong, whice results in the protease being susceptible to STI.

In general, the inhibitory effects of the different protease inhibitors on the protease activities, and consequently, on the development of *P. xylostella* were varied greatly. During larval feeding period, the levels of inhibition for all the inhibitors varied, reflecting the complexity of the *P. xylostella* midgut protease profile. During the experiment, the protease activities of all the inhibitor treatment groups changed. The inhibition level of STI on the four proteases (total protease, high-alkaline trypsin, low-alkaline trypsin and chymotrypsin) was greater than that of the other three inhibitors. STI is a natural trypsin inhibitor, which has been reported to decrease the weight of *Spodoptera litura* Fabricius (Lepidoptera: Noctuidae) larvae and pupae, delay their growth, and prolong their generation time, with these inhibitory effects being more significant in the early developmental stages (Wu et al., 2013). We found that STI had obvious inhibitory effects on *P. xylostella* larvae, which is consistent with those reported by Wang and Qin (1996) and Upadhyay and Chandrashekar (2012), showing that the greater the inhibitor concentration, the more obvious is the inhibitory effect. On feeding protease inhibitors to larvae, the insects often change their intestinal proteases to adapt to the inhibitory effects, causing a change in protease activity. When these insect larvae ingested low concentrations of protease inhibitors for long period, the activity of chymotrypsin increased, that of high-alkaline trypsin activity decreased, whereas that of total protease activity remained the same. If larvae ingested high concentrations of the protease inhibitors for long period, then the total protease and high-alkaline trypsin activities decreased (Wang et al., 1995). Protease inhibitors can result in anti-nutritional effects and break the balance among the proteases, causing a disorder in the digestive system, which would affect the growth and reproduction of the insects (Chougule et al., 2005). Oppert (1999) found that for the coleopteran pest, *Tribolium castaneum* Herbst (Coleoptera: Tenebrionidae), the control was more effective when both serine and cysteine protease inhibitors were used simultaneously. In the current experiment, changes were observed in the midgut protease activity of *P. xylostella* after it ingested the protease inhibitors for only a short period. In future experiments, the feeding time should be further extended to explore the changes in protease activity due to long-term ingestion of protease inhibitors.

Protease inhibitors have an inhibitory or activating function on serine protease in the midgut of insects, destroying coordination among the proteases which disrupt the digestion process of the insect. This affects their growth, development and reproduction (Lawrence and Koundal, 2002; Giri et al., 2005). It is also reported that in cowpea bruchid, *Callosobruchus maculatus* Fabricius (Coleoptera: Bruchidae), protease inhibitors degrade the proteases (Zhu-Salzman et al., 2003). The growth and development period of *P. xylostella* that ingested different protease inhibitors were significantly different. The larva-adult treatment durations in the protease inhibitor treatments were longer than those in the control treatments. The inhibitors delayed the growth of *P. xylostella*. The results of this treatment were the same as those reported by Ortego et al. (1998), who reported that different inhibitors had different inhibition levels for the growth of *Aubeonymus mariaefranciscae* Roudier (Coleoptera: Curculionidae). The growth and development periods were affected by the concentration of the inhibitor, wherein treatment with higher STI concentrations had a longer duration and lower survival rate than that with the other inhibitors used in this study. At STI concentrations of 0, 10, 50, and 100 μg/mL, the pupal duration, pupal weight, and emergence rate showed no significant differences, suggesting that the concentration discrepancies in concentration were minimal. However, insects had certain ability for adaptation, when STI was fed to *Prodenia litura* Fabricius (Lepidoptera Noctuidae) through multiple generations, the inhibitory effect of STI on larval weight and pupal weight was reduced and the generation period was shortened (Ogunlabi and Agboola, 2007; Opitz and Mvller, 2009). The adaptive capacity of the insect to STI was improved by regulating the growth and development process, and thus, its inhibitory effect was gradually decreased (Chougule et al., 2005; Wu et al., 2013). Protease inhibitors can suppress the activities of the larval insect midgut protease, then disrupt the digestive system and delay the growth of insects. The same result was observed in previous studies (Tamhane et al., 2005; Bhattacharyya et al., 2007; Zhu et al., 2007). Several studies have focused on efficient inhibitors that can be used for pest control, and on their usage-pattern (Macedo et al., 2002; Oliveria et al., 2007). When bean flour containing a protease inhibitor was mixed with several insecticides, the insecticidal effect was doubled, thus protease inhibitors have significant potential as synergists for new pesticides (Hou et al., 2004).

## Conclusion and Future Perspectives

In this study, we examined the effect of protease inhibitors on protease activities and on the growth and development of *P. xylostella* larvae, both *in vitro* and *in vivo* when larvae ingested protease inhibitors for a given period of time. We observed that protease inhibitors had varying degrees of inhibitory effect on protease activities and consequently, extended the growth and development periods of *P. xylostella.* Next possible step could be to use protease inhibitors as a new biological pesticides for controlling *P. xylostella* or to transfer the gene(s) of protease inhibitors to host plant which would reduce the feeding of *P. xylostella* on the plants, suppress its development, and minimize damage to the host plant.

## Author Contributions

AZ participated in the design of the study, performed the experiments and data analysis, generated the figures and tables, and helped to draft the manuscript. YinL and CL helped AZ to performed the experiments. YipL and PW conceived the study, designed the study, coordinated the study, and drafted the manuscript. All the authors approved the final manuscript.

## Conflict of Interest Statement

The authors declare that the research was conducted in the absence of any commercial or financial relationships that could be construed as a potential conflict of interest.
